# Predicting Species Diversity of Benthic Communities within Turbid Nearshore Using Full-Waveform Bathymetric LiDAR and Machine Learners

**DOI:** 10.1371/journal.pone.0021265

**Published:** 2011-06-20

**Authors:** Antoine Collin, Phillippe Archambault, Bernard Long

**Affiliations:** 1 Department of Geosciences, INRS-ETE, University of Québec, Québec, Canada; 2 Institut des Sciences de la Mer, Université du Québec à Rimouski, Rimouski, Canada; Institute of Marine Research, Norway

## Abstract

Epi-macrobenthic species richness, abundance and composition are linked with type, assemblage and structural complexity of seabed habitat within coastal ecosystems. However, the evaluation of these habitats is highly hindered by limitations related to both waterborne surveys (slow acquisition, shallow water and low reactivity) and water clarity (turbid for most coastal areas). Substratum type/diversity and bathymetric features were elucidated using a supervised method applied to airborne bathymetric LiDAR waveforms over Saint-Siméon–Bonaventure's nearshore area (Gulf of Saint-Lawrence, Québec, Canada). High-resolution underwater photographs were taken at three hundred stations across an 8-km^2^ study area. Seven models based upon state-of-the-art machine learning techniques such as Naïve Bayes, Regression Tree, Classification Tree, C 4.5, Random Forest, Support Vector Machine, and CN2 learners were tested for predicting eight epi-macrobenthic species diversity metrics as a function of the class number. The Random Forest outperformed other models with a three-discretized Simpson index applied to epi-macrobenthic communities, explaining 69% (Classification Accuracy) of its variability by mean bathymetry, time range and skewness derived from the LiDAR waveform. Corroborating marine ecological theory, areas with low Simpson epi-macrobenthic diversity responded to low water depths, high skewness and time range, whereas higher Simpson diversity relied upon deeper bottoms (correlated with stronger hydrodynamics) and low skewness and time range. The degree of species heterogeneity was therefore positively linked with the degree of the structural complexity of the benthic cover. This work underpins that fully exploited bathymetric LiDAR (not only bathymetrically derived by-products), coupled with proficient machine learner, is able to rapidly predict habitat characteristics at a spatial resolution relevant to epi-macrobenthos diversity, ranging from clear to turbid waters. This method might serve both to nurture marine ecological theory and to manage areas with high species heterogeneity where navigation is hazardous and water clarity opaque to passive optical sensors.

## Introduction

The biodiversity conservation strategy usually starts with the characterization of landscapes and biological communities, then focuses on habitats that support biodiversity positive anomalies [1]. Ecological theory assumes that species distributions are determined in part by environmental gradients and resources [2]. Defining a set of environmental variables which are recognized to entail direct or indirect responses from presence/absence species and linking them by an ecologically-relevant statistical model enable the acquisition of significant information aimed at conservation planning [2], [3], [4]. Therefore, spatial predictions of suitable habitats for hotspots represent a proactive tool for decision makers in the selection and evaluation of protected areas [5], [6]. Despite the rapidly growing use of ecological spatial modelling within last decade [3], [6], [7], [8], [9], Austin [2] showed a paucity of consistency between species predictive models and ecological theory as well as little discussion about these discrepancies. Statistical models based on linear regression such as Generalized Linear Models (GLM), Multivariate Adaptative Regression Splines (MARS) or Structural Equation Modelling (SEM) are extensively used while being constrained by assumptions that ecological data is unlikely to meet, such as normality and homoscedasticity in species' response [2], [3]. However, new approaches calling upon nonparametric coefficients, especially decision trees, have been demonstrated to outperform linear models since both linear and nonlinear relationships between biotic and abiotic components are well identified [6], [10]. Pittman et al. [6] equated three modelling techniques for predicting fish species richness across shallow-water seascapes and concluded that the tree-based model was more proficient than multiple linear regression and neural networks, showing an overall map accuracy of 75% for osteichthyes.

Traditional methods carried out to evaluate and conserve marine biodiversity have mostly been supported by punctual and scattered data derived from cursory surveys [11], likely hindering accurate predictions of areas that should be sanctuarized. In addition to the development of improved analysis techniques, two major advances have occurred during the last ten years and have allowed to address marine conservation issues: the progress of remote sensing and the change of biological level. Firstly, despite the relative opacity of water regarding electromagnetic waves, recent improvements have enabled the acquisition of quasi-continuous measurements of environmental predictors, either proximal or distal, over broad seascapes and conduct species distribution or richness modelling through acoustic methods and optical remote sensing over clear water [6], [7], [12], [13]. Secondly, albeit usually the core of a conservation perspective, a species-level approach is not appropriate for direct monitoring by either the best echo-sounder or even the most valuable, spectrally and spatially, spaceborne sensor. Adopting habitat-level surrogates for underwater biodiversity both interfaces the resolution inherent to the sensor with the size of the measured biotic unit and promotes the conservation of common species complementary to species of interest (e.g., keystone, umbrella or rare) which would not have received much consideration otherwise [11].

Depth and structural complexity are indirect environmental gradients which play fundamental roles in macrobenthic and fish species richness and abundance [6], [14], [15]. This assertion is especially intuitive for epi-macrobenthic species, whose adults are sessile and communities strongly influenced by habitat selection preferences of larvae, sensitive to depth-related temperature, pressure, salinity and bottom structure [16]. Structurally-complex habitats present larger surfaces than smooth ones, constitute larger targets for potential colonizers, and can accordingly receive more immigrants [17]. Robust predictions of species features are closely dependent on the ability to match response data resolution with more proximal predictors ( i.e. to accurately monitor seafloor physical heterogeneity). Austin [2], [4] duly advocated that response data resolution should be consistent with ecological theory and the predictors being used. However, spaceborne sensors do not offer high enough resolution to provide ecologically relevant structural information. Optically passive airborne platforms are able to derive such resolution but are limited by water clarity, whereas waterborne devices, unrestricted by turbidity, cannot acquire data over shallow and hazardous (strong currents and wind) waters [18].

The airborne bathymetric LiDAR (Light Detection And Ranging) is the only technique capable of providing large datasets over turbid and shallow water at an appropriate metricresolutionto explain the spatial distribution of abiotic and biotic components. The bathymetric LiDAR is an active laser-based pulsed altimeter that allows for accurate depth measurements (up to three times the Secchi depth) by generating Digital Depth Models (DDM) during optimal environmental windows, making it a time and cost-effective tool in support of coastal hydrography [18]. LiDAR has recently been used towards benthic habitat mapping [15], [19], [20], [21], as well as the characterization of their structural complexity using full-waveform data [22]. Using such an innovative technique to derive seabed and bathymetric features in modelling species richness, abundance and composition in opaque nearshore areas is a novel approach and may considerably facilitate the delineation of areas showing high levels of ecological importance.

This work's objectives are to (1) establish the species richness, abundance and composition of the epi-macrobenthic species within the Baie des Chaleurs (Québec, Canada) , (2) retrieve seabed type and diversity data as well as morphometric features from full-waveform LiDAR data, (3) assess the amount of variability in biotic indices that can be reliably predicted by LiDAR-derived controlling factors by means of machine learning techniques, and (4) model the spatial patterns of biotic information across the LiDAR-surveyed whole area using GIS.

## Materials and Methods

### Study site

**Figure 1:**
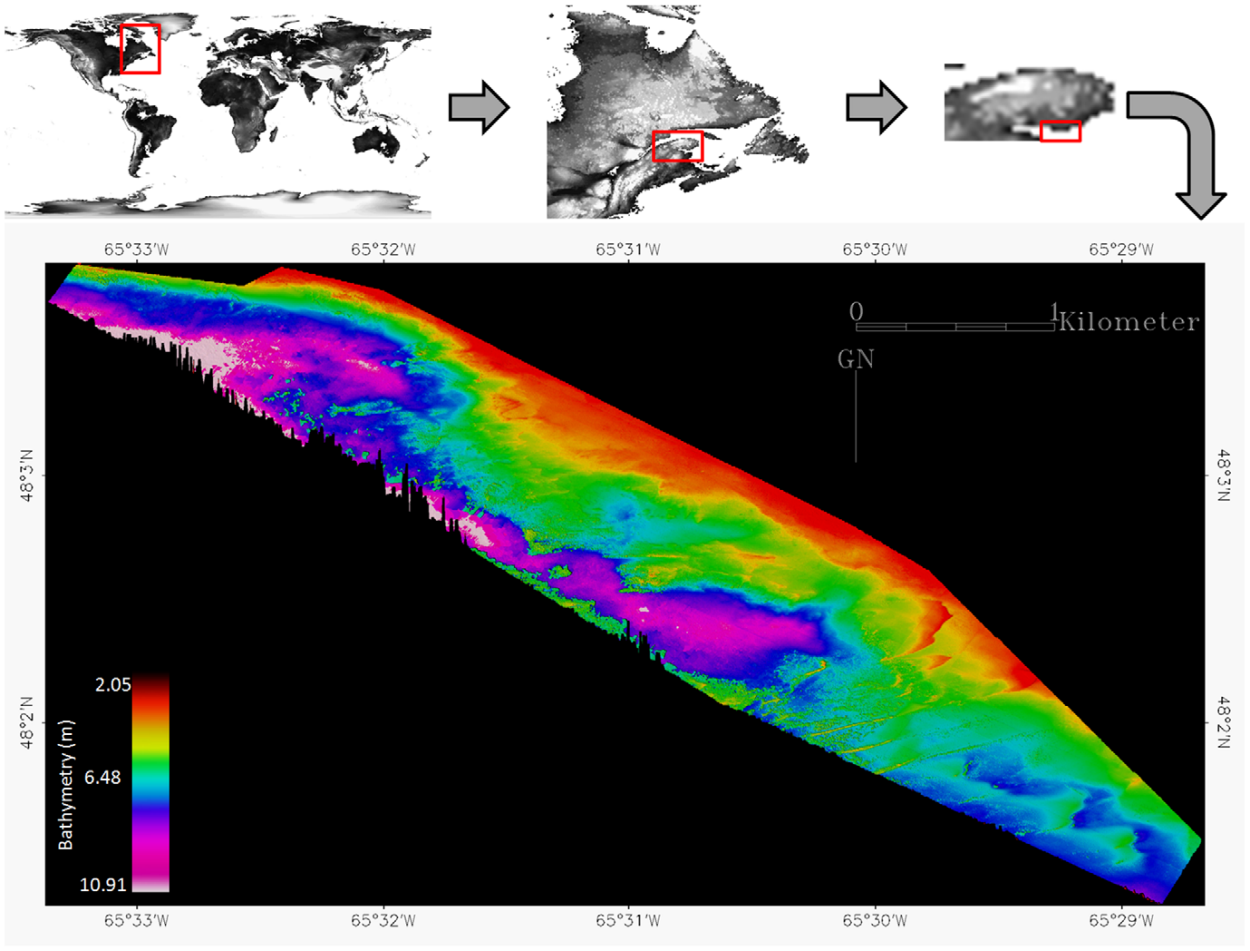
Location of the study area along Bonaventure's nearshore area (South of Gapsesia Peninsula, Quebec, Canada) and the underlying LiDAR bathymetric map based upon a 2 m grid. Water depths ranged from 2.05 to 10.91 m.

The study took place along a part of the north shore in the Baie des Chaleurs (48°N, 65°W), southern Gulf of Saint-Lawrence, Quebec, Canada (Fig. 1). The Baie des Chaleurs is a semi-enclosed basin 130 km long, steadily widening and deepening towards the opening, i.e., 40 km and 90 m, respectively. The coast of Saint-Siméon – Bonaventure was selected based upon the broad range of habitat diversity: a river delta, sand banks, boulders, pebbles, kelp fields and an eelgrass meadow. The structural complexity inherent to boulders and their surroundings offer refuges, whereas the sedimentary crown is a suitable hunting-ground for lobsters. The alternation and the adjacency of algae-covered boulders and sand patches create a heterogeneous mosaic in this benthoscape, and are therefore correlated with a high probability of lobster (*Homarus americanus*) presence, as demonstrated by the monospecies-oriented catch effort. Two spionidae polychaetes, *Prionospio steenstrupi* and *Spiophanes bombyx*, the bivalve *Spisula* spp., the crustacean *Corophium bonelli*, and the echinoderm *Echinarachnius parma* were sampled over sandy-gravely seabed, while the macroalgae *Laminaria* spp. populated cobble and pebble bottoms [23].

### Epi-macrobenthic diversity and abundance responses

The ecological model assumed that surveyed epi-macrobenthic species distributions were determined at least in part by environmental gradients, and that reasonable approximations for these gradients could be estimated [2], [4]. Despite the added theoretical complexity, collective properties of the epi-macrobenthic populations, such as species richness, abundance and composition, were investigated owing to their ecological significance.

### Sampling design

Macrobenthic sampling surveys were carried out between the 25th June and the 3rd July 2006, with an underwater digital high-resolution (5 megapixels) camcorder. The camera was mounted on a tetrapod frame, and dropped over the seabed. Face to the field of view, a reference ruler was added on the frame in order to evaluate the size of objects of the seafloor. Each image was captured from a continuous video and monitored 0.16 m2 (0.4×0.4 m). A total of 300 images were collected and their position accurately recorded by a Differential GPS (DGPS), meeting a submetric horizontal accuracy. Constrained by the draught-imposed limit landward and the loss of the LiDAR bottom signal seaward, which a *posteriori* corresponded to the 16 m isobath, the sampled area covered 8 km2. Point-observations were spread across the entire study area, without *a priori* knowledge of the distribution of substrata. The analysis of the seabed image allowed the computing of diversity and abundance indices at each point.

### Diversity and abundance indices

Biological diversity and abundance indices associated to epi-macrobenthic features were derived from a species-sediment matrix. Each row of this matrix corresponded to the percentage of the surface covered by epi-macrobenthos and sediment variables. The quantification of the surface occupied by each benthic component was facilitated by the superimposition of a grid of 100 uniformly distributed squares on each photograph [24]. The identification of both the lowest possible taxonomic level and the grain size constituted 12 biotic and 4 abiotic variables: Crustacea, Echinoidea, Annelida (and/or burrows), Gastropoda, Asteroidea, Dead shells, *Fucus* spp., *Zostera marina*, *Chondrus crispus*, *Laminaria* spp., *Chorda tomentosa*, *Polysiphonia* spp., Boulders >256 mm with encrusting algae, Cobbles>64 mm, Pebbles>4 mm, Fine-sand>0.06 mm.

The α-diversity was defined as the species richness (S) of epi-macrobenthos within each station. Since the observed number of species in the survey is a biased estimator of the true species richness in the area, and since the observed species number increases non-linearly with sampling effort, the species richness of the observed epi-macrobenthic species richness was referred to as species density (d) per m2. The abundance and composition of biotic components were examined by means of seven indices: the overall abundance (A), i.e., sum of the percentages of the colonized surface, and its log transformation, the Simpson diversity index applied to the percentages of the surface (D), expressed as one minus the sum of the squares of the ratio of the image covered by the ith biotic variable with overall abundance, and its log transformation, the Shannon diversity index applied to the percentages of the surface (H'), corresponding to the opposite of the sum of the fraction of the image covered by the ith biotic variable multiplied by the natural logarithm of this same fraction, and its log (X+1) transformation, as well as a modified Pielou evenness (mJ'), computing the ratio between the previous Shannon index and the natural logarithm of the species richness +1.

The data model used in this work was clearly underpinned by both the purpose of the paper and the scale, which is consistent with the underlying ecological theory. In addition, the selection of biotic variables, namely various measures of diversity and abundance data, was incorporated within the data model. The last salient part of the data model relied upon the selection of environmental variables. Albeit indirect, the bathymetry and derived features, as well as the seafloor nature and diversity, were recognized to be robust proxies for modelling spatial distribution of benthic species [5].

### Seafloor structure and diversity predictors

The bathymetric variability and related spatial configuration structuring the neritic benthoscape at fine-scale as well as the seafloor structure and diversity were quantified using the Scanning Hydrographic Operational Airborne LiDAR Survey (SHOALS) system.

### Bathymetric LiDAR

**Figure 2:**
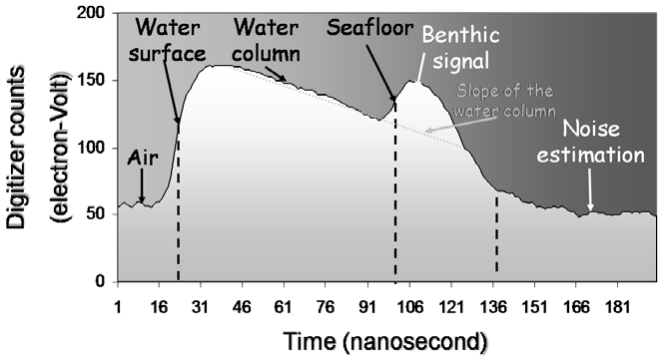
Chart of the bathymetric LiDAR full-waveform monitored by the green (532 nm) channel. This signal was acquired at 4.50 m depth and the oblique dashed line consisted of a linear fit of the water column return.

The SHOALS system is an airborne, scanning, pulse-based laser altimeter that measures accurately and reliably the distance from the aircraft to the surface and the sea bottom. Unlike passive sensors constrained by solar irradiance, the active bathymetric LiDAR has the capabilities to acquire bathymetry and seafloor data in only accounting for its own energy source, thus providing data over water bodies considered too turbid for any optical passive sensors. Other conspicuous reasons for integrating this state-of-the-art technology to marine ecology are i) the ability to complete rapid high-resolution surveys over large areas in very short periods (relevant for monitoring seasonal or storm-induced change), increasing the cost-effectiveness [18], ii) the capacity to map habitats where it would be hazardous to use waterborne techniques [25], and iii) the facility to simultaneously survey the subtidal, intertidal and supratidal ecological structures [26]. The SHOALS measurements are accurate to IHO Order 1 standards, i.e., ±0.15 m vertically and ±2 m horizontally [25]. Each sounding was precisely positioned by the recording of the aircraft (using a differential and kinematic GPS) and its roll, pitch, and yaw at 200 Hz by an Inertial Measurement Unit (IMU). The system emits the 532 nm wavelength (green) from a Nd-YAG laser, allowing the detection of bottom characteristics because of its high water transmission. In addition, the SHOALS system is a full-waveform system which allows the digitization and recording of the entire backscattered signal as a function of time for each laser pulse. A typical waveform may be divided into three distinguished parts: water surface, water column, and the benthic return (Figure 2). The SHOALS scanning operated at 3 KHz, and the study area was covered by a series of 8 NW-SE overlapping flight lines at 269.3±9.6 m altitude inducing a swath width of 198.1±4.4 m and a spot spacing of 2 m, i.e., 2 548 536 soundings covering a 7.982 km2 seafloor area. The maximum depth penetration matched the 16 m isobath owing to water clarity of this bay at this time.

### Bathymetry and derived morphometric indices

The influence of the bathymetry and related structural complexity on benthic community ecology has been well documented [27], and is what prompted the use of these continuous variables as robust environmental predictors. The bathymetric LiDAR points, containing easting, northing and ellipsoidal height, were processed into IDL-ENVI 4.2 [28] so that different surfaces were constructed. Triangular Irregular Networks (TIN) were constructed based on the ellipsoid-corrected height that was linearly interpolated to a 2 m resolution Digital Depth Model (DDM) with a very high resolution accuracy of 15 cm. In addition to the mean bathymetry, the complexity of the benthic structure was examined in extracting 12 bathymetric features, such as Absolute Roughness, Local Roughness, Slope, Aspect, Shaded Relief, Profile Convexity, Plan Convexity, Longitudinal Convexity, Cross Sectional Convexity, Minimum Curvature, Maximum Curvature, Root Mean Square Error (Table 1). Each morphometric index of the fine-grained benthoscape was quantified and then spatially rendered by building a 2 m grid. Based upon these macrohabitat-related measurements, two bathymetric feature classification images were created at two spatial scales (6 m and 12 m), and each pixel of the output was assigned according to the following terrain types: peak, ridge, pass, plane, channel or pit [29].

**Table 1:** Description of the LiDAR-derived environmental predictors.

Predictors		Description	Unit
Bathymetry	Bathymetry	Average bathymetry	Meters
	Absolute Roughness	Standard Deviation of bathymetric values between pixel and eight neighbours	Meters
	Local Roughness	Slope-corrected Absolute Roughness	Meters
	Slope	Average rate of change in slope between pixel and eight neighbours	Degrees
	Aspect	Horizontal direction to which the pixel slope faces	Degrees
	Shaded relief	Computed cast shadow thrown upon raised bathymetric DEM (Lambertian surfaces)	Lux [0-1]
	Profile Convexity	Intersection between the plane of the bathymetry axis and aspect direction (rate of change of slope along the profile)	+:convex-:concave
	Plan Convexity	Intersection between the latitutde-longitude (rate of change of aspect along the plan)	+:convex-:concave
	Longitudinal Convexity	Intersection between the plane of the slope normal and aspect direction	Degrees
	Cross-sectional Convexity	Intersection between the plane of the slope normal and perpendicular aspect direction	Degrees
	Minimum Curvature	Minimum overall surface curvature	Degrees
	Maximum Curvature	Maximum overall surface curvature	Degrees
	Root Mean Square Error	Residuals between the quadratic surface and the actual digital elevation data	Meters
	Bathymetric features (6 m)	Classification of morphometric features at 6 m scale: ridge, pass, channel	Class
	Bathymetric features (12 m)	Classification of morphometric features at 12 m scale: ridge, pass, channel	Class
Substratum	Substratum type	Classification of substratum type relied upon 16 waveform-based statistics and camera-driven photos	Class
	Substratum Dissimilarity	Bray-Curtis Dissimilarity applied to substratum type map	Percent
	Substratum Evenness	Pielou Evenness applied to substratum type map	[0–1]
Benthic Waveform	Mean	Mean of the probability distribution of the benthic waveform intensity	Photo counts
	Variance	Squared deviation of the probability distribution of the benthic waveform intensity	Photo counts
	Skewness	Asymmetry of the probability distribution of the benthic waveform intensity	Photo counts
	Kurtosis	Flattening of the probability distribution of the benthic waveform intensity	Photo counts
	Median	Median of the benthic waveform intensity	Photo counts
	Mean Absolute deviation	Average deviation from the Mean	Photo counts
	Minimum	Minimum value of the benthic waveform intensity	Photo counts
	Maximum	Maximum value of the benthic waveform intensity	Photo counts
	Area Under Curve	Integration of the tabulated waveform on the closed benthic interval	Photo counts^2^
	Intensity Range	Intensity Difference between the maximum and the minimum values of the benthic waveform	Photo counts
	Time Range	Time Difference between the maximum and the minimum values of the benthic waveform	Nano-second
	Intensity Shannon	Shannon diversity index applied to benthic waveform intensity deciles	[0–1]
Transition Waveform	Mean	Mean of the probability distribution of the water column-end/benthic-start waveform intensity	Photo counts
	Variance	Squared deviation of the probability distribution of the water column-end/benthic-start waveform intensity	Photo counts
	Skewness	Asymmetry of the probability distribution of the water column-end/benthic-start waveform intensity	Photo counts
	Kurtosis	Flattening of the probability distribution of the water column-end/benthic-start waveform intensity	Photo counts

### Seafloor nature

The seafloor nature may be deemed as habitat type in this study's context. This nominal environmental predictor has long been linked to macrobenthic assemblages [30], [31], [32]. The bathymetric LiDAR has recently been used to discriminate benthic habitats through waveform-retrieved statistical parameters from the benthic part [20]. Briefly, the designed methodology consisted of retrieving the benthic waveform, computing 12 statistics, non-linearly regressing them on depth, keeping the first Principal Components (PC) and classifying them (detailed in [20]). The same authors then added the quantiles of the benthic waveform and tested the enhanced LiDAR-derived dataset as a surrogate for the benthic type [22]. We proposed here to refine the methodology in synthesizing the deciles' information, 10 intensity-related bands, into a band corresponding to the Shannon diversity index.

A greater diversity of deciles within the benthic waveform returned a high *H'dec* value. In addition to the *H'dec* variable, other descriptive statistical variables were computed - namely mean, variance, skewness, kurtosis, median, mean absolute deviation, area under curve, maximum, minimum, time and intensity ranges between 0% and 100% of the benthic waveform, as well as mean, variance, skewness, kurtosis, quantifying the curve stretching from the end of the water column to the start of the benthic signal. The dataset, composed of 16 variables and 2548536 soundings was then constrained by the methodology designed by Collin et al. [20], [22]. A 2 m×2 m seafloor map was achieved using a supervised classification, performed by the Support Vector Machine (SVM). Given that LiDAR soundings were accurately located to within 2 m, no post-classification resampling was proceeded. Accuracy assessment of the seafloor map was conducted based on a confusion matrix confronting the resulting map, derived from training polygons (2/3 ground-truth pixels), and validation polygons (1/3 ground-truth pixels).

### Seafloor diversity

In addition to the seafloor categorization, the seafloor β-diversity was calculated using special codes written in IDL 6.2 [28]. Applied to the thematic seafloor map (raster grid of pixels), two algorithms returned the composition of seafloor types, in the form of metrics, around one pixel within a kernel of a given radius.

The Bray-Curtis dissimilarity [33] value reflected the dissimilarity between the species compositions of two locations. A score near 0 was obtained between similar regions, while a maximum score of 100 described two entirely different locations. The Pielou evenness index [7] was comprised into 0-1 boundaries, where 0 meant a single substratum type covering the kernel, and 1 referred to an equal proportion of all substratum types within the kernel.

### Machine learners

**Figure 3:**
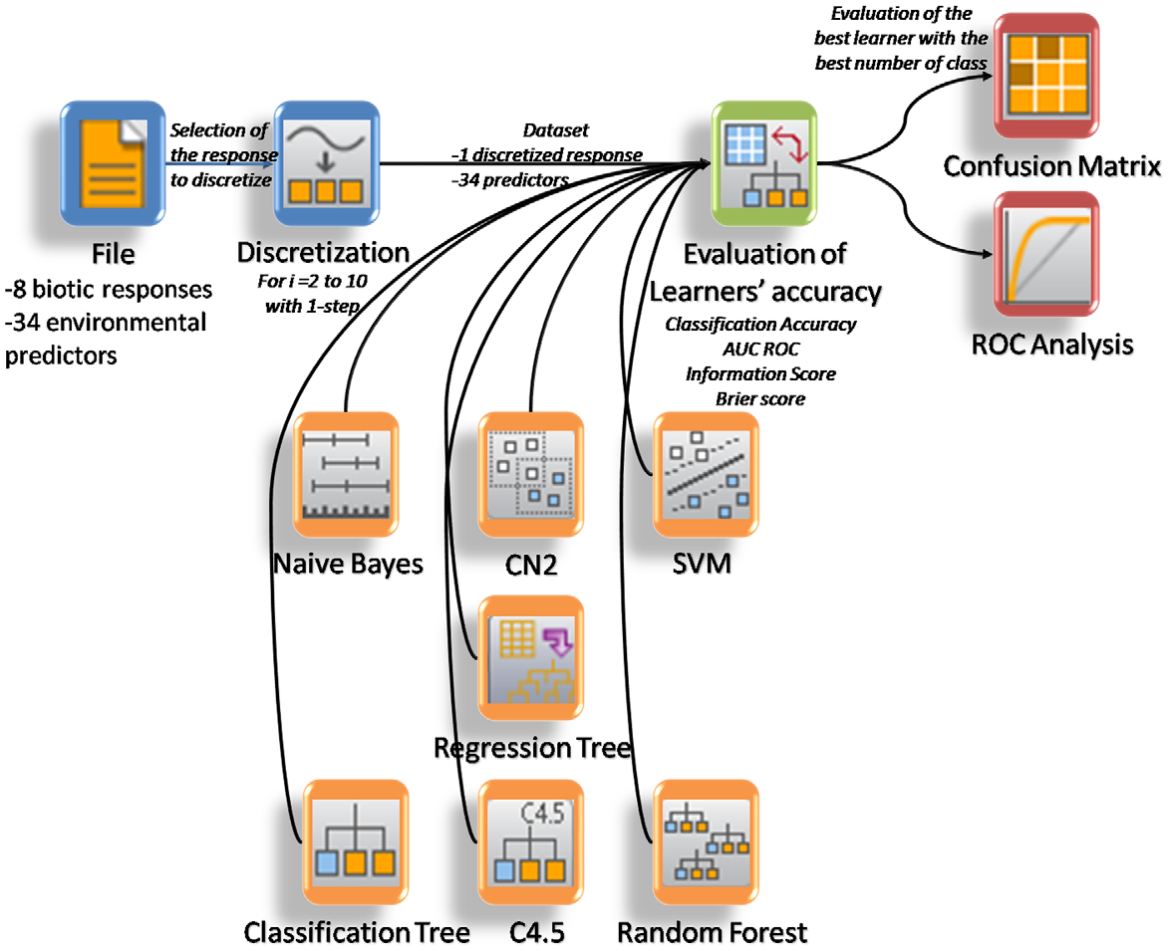
Workflow summarizing the statistical analysis. Blue tabs indicated initial datasets and the discretization procedure; orange tabs highlighted machine learners used; green tab represented the models' evaluators and red tabs represented analytical evaluators.

Habitat suitability and species distribution predictions require reliable and robust modelling techniques. Because of the widespread variety of interactions between species individuals with their biotic and abiotic environment, it is well admitted that traditional statistical models mostly provide relationships between the contextual realized niche of a species rather than the fundamental one [3], [34], [35]. However, recent research advancements in statistics, called machine learning, enable to overcome overly simplistic species-habitat modelling by automatically recognizing complex patterns based upon improvements and in making decisions based on these improvements [36]. In addition to being clearer in terms of ecological interpretation (decision rules), supervised learners can elucidate very complex underlying relationships even though the mathematical form of the dependencies are unknown. Thus, their systematic superiority of performance over linear models is even generalized [10]. Model performance, however, has commonly been assessed by a single n-class combination, which might lead to questionable outputs. While developing a series of seven statistical models, such as Naïve Bayes, Regression Tree, Classification Tree, C 4.5, Random Forest, Support Vector Machine, and CN2 learners, we analyzed their performance against the number of categories (or classes) within each of the eight biotic responses (Figure 3).

The Naïve Bayes is a simple probabilistic classifier based on applying Bayes' theorem with strong independence assumptions. Relative frequency was the method used for estimating prior class probabilities from the data, and the classification threshold was fixed at 0.5.

Decision trees are constituted of leaves representing predicted outcomes and branches representing conjunctions of features that lead to those outcomes [37]. The Regression Tree gives a numeric (e.g., real number) value as the output, and is built by using a data-analysis method that most effectively and recursively partitions data into sets each of which are simply modeled using regression methods. Settings specified to the regressor were the binarization, i.e., the splitting of values within multivalued variables into two groups in the particular node, the pruning during the induction, i.e., prohibiting the splitting with less samples than 10, and finally, the pruning after the induction with 5-estimate of error. Unlike Tree Regression, leaves of Classification Tree are composed of classes (categorical values) to which data belong. However, settings assigned to Classification Tree were rigorously similar to those of Regression Tree, with the addition of a variable selection criterion based upon the information gain (difference in entropy) as well as a recursive merge of leaves with same majority class within the post-pruning. Overfitting of decision trees can thus be avoided by halting tree growth when no more significant information can be gained. The originality of the C 4.5 algorithm relies on the gain ratio (variable selection criterion) that results from choosing the variable with the highest gain ratio for splitting the data and making the decision [38]. The pre and post-prunings were defined by 10 samples minimum for splitting and a confidence level of 25, respectively. Random Forest is a classifier that builds a set of classification trees [37]. Each tree growth is developed from a bootstrap sample from data, and an arbitrary (or randomly) subset of variables (n = 6, i.e., square-root of the number of predictors) from which the best variable regarding splitting is adopted. The classification is founded on the majority vote from individually developed tree learners in the forest (constituted, here, of 10 trees). Although Brieman [37] advocated letting trees grow without any pre-pruning, we set at 10 the minimum number of samples within a node before splitting, for the sake of unequivocal comparisons. The Support Vector Machine classifier constructs a separating hyperplane or set of hyperplanes in the variable-dimensioned space which has the largest distance to the nearest samples of different classes. In order to create non-linear classifiers, we chose a Radial Basis Function kernel that efficiently (less complex and fewer numerical difficulties than a polynomial kernel) transforms variable space to a new feature space supporting the margin-maximized hyperplane [39]. Furthermore, even if it slowed down the algorithm, the dataset was normalized for better classification performance. The CN2 classifier produces an ordered list of if-then rules from data. Rules induced by CN2 each have the form “if *complex* then predict *class*”, where *complex* is the conjunction of variable tests [40]. The iteratively-searched complex was optimally both predictive and reliable according to Laplace's evaluation function [41]. Carrying out a pre-pruned general-to-specific search, we set two parameters of Likelihood Ratio Statistics, namely Alpha ( = 0.05) and Stopping Alpha ( = 0.2). Alpha determined required significance of a rule when compared to the default rule, and Stopping Alpha verified whether the last specialization of the rule was significant enough. The best rules, whose number was set at 5, were, in each step, further specialized, while other rules were discarded. Finally, the algorithm was implemented so that all covered samples were removed and learning on remaining samples was continued (i.e., exclusive covering).

### Spatial autocorrelation

Spatial autocorrelations of the selected diversity response (initial and predicted) were evaluated using the univariate Moran's I. The Moran's I looks for an overall pattern between the proximity and similarity of samples, in comparing the differences between neighboring samples and the mean to provide a measure of local homogeneity. This index indicates high positive and negative spatial autocorrelation when tending to 1 and −1, respectively, while it reveals that data are spatially uncorrelated when near 0. Moran's I was computed using the open-source GeoDa 0.9.5 software intended for exploratory spatial data analysis [42].

### Evaluation of learners' accuracy

Predictions of the 8 biotic responses stemming from the 7 learners were evaluated by means of a 34%-random test dataset (n = 100), as well as 4 performance scores. We chose a high dataset size (relatively to the training dataset) in order to avoid overestimated performance measures, as smaller size, cross-validation or leave-one-out methods would run. The Classification Accuracy (CA) of a learner is the ratio of the number of samples correctly classified (true positives + true negatives) divided by the total number of sample cases. As a summary statistic, the Area Under receiver-operating characteristics Curve (AUC) was equal to the probability that a learner ranked a randomly-chosen positive sample higher than a randomly-chosen negative one. A high-quality test had an AUC approaching 1. The Information Score (IS), i.e., information-based evaluation criterion, was the average amount of information per classified sample, as defined by Kononenko and Bratko [43]. It excluded the influence of prior probabilities (which might enable a completely uninformed learner to trivially achieve high classification accuracy), and dealt with various types of imperfect or probabilistic answers. The IS's upper limit equaled to the entropy (best) of the class and the lower limit is 0 (worse). Brier score measured the accuracy of probability assessments, which measured the average deviation between the predicted probabilities of events and the actual events. The Brier score was a proper score function (0: perfect prediction, and 1: completely false prediction) that measured the accuracy of a set of probability assessments, which computed the average squared deviation between predicted probabilities of events and the actual events [44]. In addition to calculations of those performance measures, we tested the evolution of the learners' predictions in respect to the number of responses' class. Based upon the equal-width discretization process of the response variable [41], performance accuracy was evaluated for 2 to 10 classes with a 1-class step.

The confusion matrix and the Receiver Operating Characteristics (ROC) were exclusively employed for the best learner with the best number of class, and allowed to support finer assessments of performance.

The statistical distributions for each biotic response was analyzed with the software JMP 8 [45], and models were developed using the open-source Orange software [41].

## Results

### Biological responses

**Figure 4:**
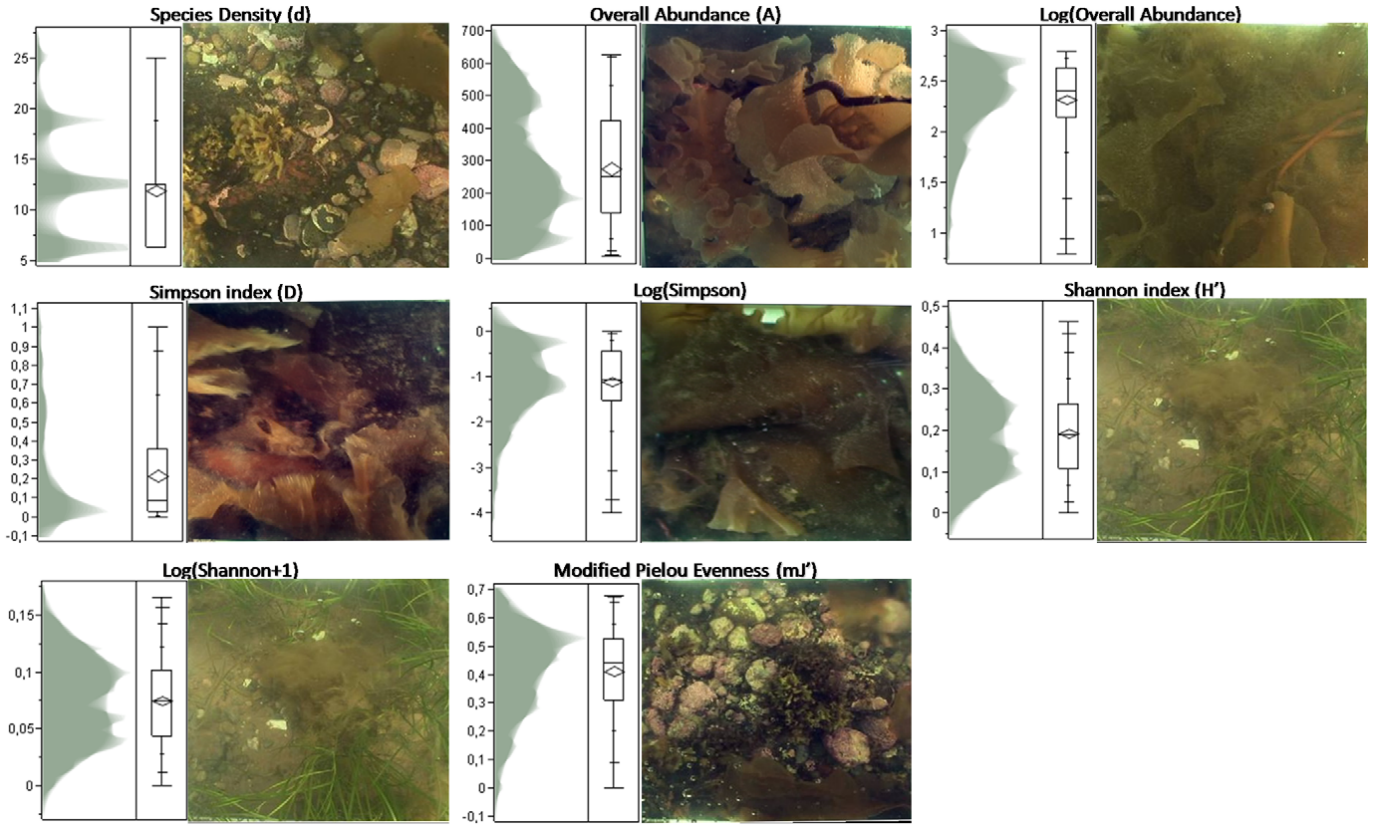
Distributions of the eight biotic indices in the form of shadowgrams statistically analyzed by quantile box plots, and augmented by the photograph of the station corresponding to the maximum of the related index.

The eight biotic indices showed both uni- and multimodal Gaussian distributions (Figure 4 and Table 2). As a categorical index, Species Density (d) displayed the most well-defined multimodal distribution, i.e., a fourfold one. This index was significantly correlated with Shannon index (H'), 0.8322 with p<0.05, as well as Log(Shannon+1), i.e., Log(H'+1), 0.8262 with p<0.05. The abundance appraisal, Overall Abundance (A), might be depicted as tri- or bimodal distributions. The distribution of its Log transformation (Log(A)) was much closer to a single leptokurtic mode with negative skew. While A was significantly correlated with Simpson index (D), 0.913 with p<0.05, and Log(Simpson), or Log(D), 0.9238 with p<0.05, Log(A) was significantly correlated with Log(D), 0.9799 with p<0.05. The Simpson index (D) distribution matched a positive-skewed leptokurtic unimode, and the distribution of its Log transformation (Log(D)) exhibited a negative-skewed leptokurtic bimode. The histogram of Shannon index (H') revealed a positive-skewed platykurtic bimode. While the pattern of Log(H'+1) distribution basically matched that of H', lowered variance and skewness characterized it. Both latter distributions were significantly correlated with modified Pielou Evenness (mJ'), 0.8772 with p<0.05 and 0.8875 with p<0.05, respectively. As for mJ', its distribution followed a strongly negative-skewed platykurtic unimode.

**Table 2:** Descriptive statistics of the eight biotic indices.

Biotic indices	Mean	Variance	Skewness	Kurtosis	Coefficient of Variation
Species Density (d)	11.83	25.68	0.58	−0.3	42.83
Overall Abundance (A)	274.83	29594.71	0.36	−0.98	62.59
Log(Overall Abundance)	2.31	0.15	−1.14	1.17	16.6
Simspon (D)	0.21	0.07	1.35	0.68	121.87
Log(Simpson)	−1.17	0.59	−0.79	0.58	−68.98
Shannon (H')	0.19	0.009	0.17	−0.8	51.48
Log(Shannon +1)	0.07	0.001	0.028	−0.86	48.1
Modified Pielou Evenness (mJ')	0.41	0.02	−0.63	−0.17	36

### Learners and evaluators

**Figure 5:**
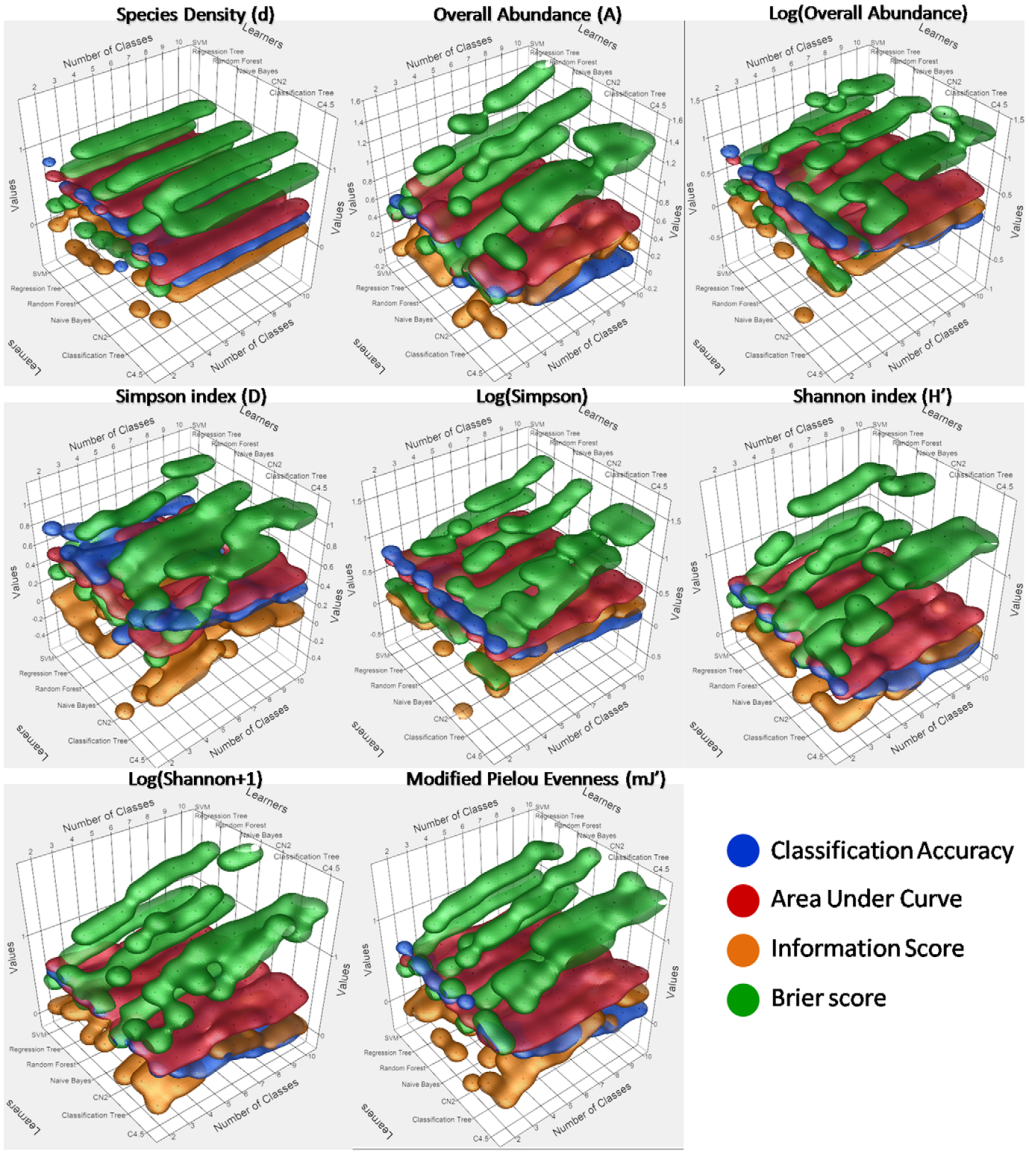
Three-dimensional scatterplots of the eight biotic indices representing the values taken by four evaluators in respect to the seven machine learners and to nine numbers of classes. The four coloured envelopes correspond to the four nonparametric density contours (each one associated with one evaluator group), drawing a 50% kernel contour shell around the points.

Along the increasing gradient of the number of classes, the four evaluators basically indicated a performance reduction for most of the learners (Figure 5). The best performance was therefore attained when the discretization was only binary (i.e., 2 classes), except for the evaluator Information Score (IS). The maximum of the Classification Accuracy (CA) met 0.83 with both the Support Vector Machine (SVM) and C4.5 while classifying Species Diversity (d) and Log(A), respectively, into 2 classes. The Area Under receiver-operating characteristics Curve (AUC) also topped with 2 classes at 0.78 resulting from the d classification by Naïve Bayes (NB). The best performance of Brier score (Bs), i.e., 0.28, also emerged with 2 classes stemming from the d classification by SVM. As for IS, the maximum, i.e., 0.56, was met with 7 classes resulting from the C4.5-clustered A. For all learners, the decrease of the CA performance across the number of classes strongly matched a number of classes-inversed model (Table 3). Although the best model depicting the decline of the AUC and the IS as a function of the number of classes was also an inverse model, the scores were lowly significant and even insignificant. Since a good prediction amounts to zero-neighboured Brier index, the loss of performance across the number of classes was satisfactorily modeled by a growing inverse adjustment, whose R2adj. reached sizeable values. Since binary classifications oversimplified the current ecological modelling, we excluded it from the selection of the best algorithm. Hence newly-highlighted maxima of CA, AUC and Bs were systematically found with the Random Forest (RF) learner related to a tercile discretization, and D (0.69), A (0.73) and D (0.49). The classification involving D with the RF and three classes have hence been chosen according to both CA and Bs indices.

**Table 3:** R^2^ adjusted of the model describing the evolution of the eight biotic indices against the inversed number of classes as a function of the four evaluators.

Biotic indices	Classification Accuracy	Area Under Curve	Information Score	Brier score
Species Density (d)	0.44	0.12	0.16	0.28
Overall Abundance (A)	0.85	0.05	0.09	0.46
Log(Overall Abundance)	0.83	0.15	0.27	0.65
Simspon (D)	0.23	−0.02	0.19	−0.51
Log(Simpson)	0.88	0.23	0.17	0.63
Shannon (H’)	0.88	0.15	−0.000045	0.36
Log(Shannon +1)	0.91	0.08	0.006	0.35
Modified Pielou Evenness (mJ')	0.87	−0.009	0.48	0.43

### Selected model

**Figure 6:**
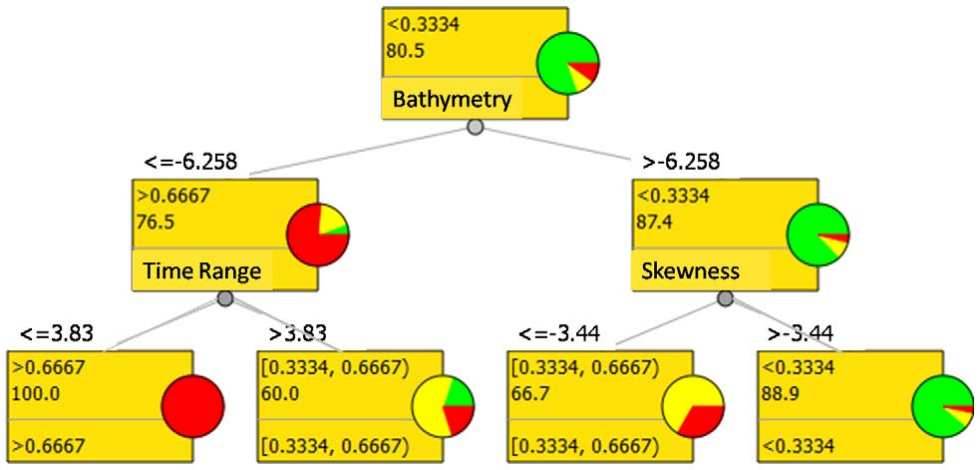
Random Forest tree model for Simpson index (D) discretized into three classes across a neritic benthoscape located north of the Baie des Chaleurs (Québec, Canada). Within each node are mentioned the label of the class (1st line), the probability of belonging to the target class (2nd line) and the splitting variable. Above the node is indicated the threshold value related to the splitting variable inherent to the previous node. Pie plots associated with each node show the number of training samples belonging to the <0.3334 (green), [0.3334, 0.6667) (yellow), and >0.6667 (red) classes.

Neither the initial Simpson diversity dataset nor modelled Simpson values displayed significant spatial autocorrelation (Moran's I = 0.13 and 0.36, respectively). Those results assumed that the contribution of the spatial autocorrelation was sufficiently low to not bias the predictions. The selected Random Forest Tree consisted of 7 nodes with 4 leaves including bathymetry, time range and skewness predictors (Figure 6); two latter ones were derived from LiDAR benthic waveform (Figure 2). The contributions of these 3 splitting variables explained 62.83% of the total variation in epi-macrobenthic Simpson diversity. The first split was based upon mean bathymetry, and the learner computed a threshold value of 6.25 m depth to discriminate deeper habitats from habitats closer to the shoreline. On one side, deeper habitats were divided into high and medium Simpson diversity (i.e., >0.6667 and [0.3334, 0.6667), respectively) according to the time range split evaluated at 3.83 ns. On the other side, shallower habitats were distinguished between medium and low Simpson diversity ([0.3334, 0.6667) and <0.3334, respectively) supporting from the splitting of the skewness variable optimized at −3.44 ns (Figure 6).

**Figure 7:**
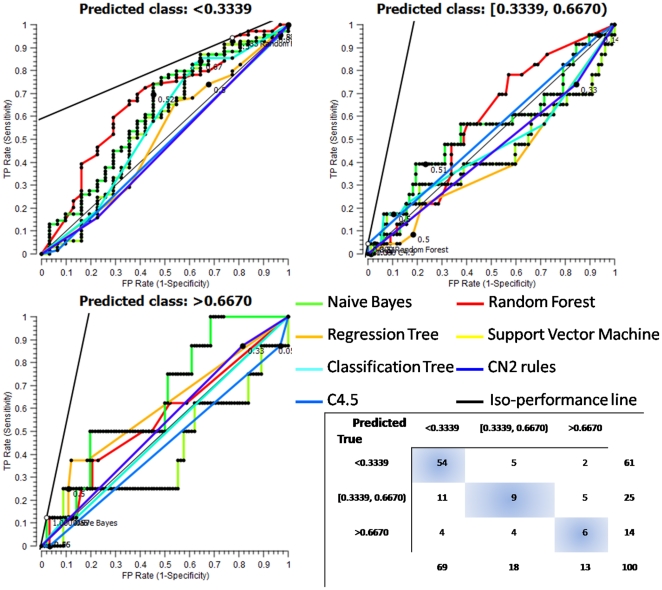
Receiver Operating Characteristics curves of the three final Simpson index (D) classes. The diagonal line (black thin) represents the behaviour of a random classifier. The iso-performance line (black bold) embodies all the points subject to trade-off between true positive (benefits) and false positive (costs), in the ROC space. The confusion matrix is depicted on the bottom right corner.

Despite the best overall performance among learners, the rate of good prediction depended upon the target class, as both confusion matrix and ROC curves demonstrated it (Figure 7). The low Simpson cluster (<0.3339) was highly satisfactorily classified (78.3% and 88.5% of predicted and true proportions, respectively) by RF as both the upper left position of the red ROC curve, relatively to other coloured curves, and the optimal threshold given 500/500 costs, indicated it. The confusion matrix summarized a moderate performance (50% and 36% of predicted and true proportions, respectively) concerning the medium Simpson class [0.3339, 0.6667). However, the RF-related envelope still encompassed the tangential point of the iso-performance line, and outperformed the other learners from a sensitivity neighbouring 0.58 to 1. Note that Regression, Classification and C4.5 classifiers displayed negative predictive power. Finally, even if the confusion matrix showed moderately-scored classification (46.1% and 42.8% of predicted and true proportions, respectively) into the high (>0.6667) Simpson class, the RF ROC curve revealed a non-random classification (above the diagonal), as well as best-emphasized convexity (distance regarding the diagonal) together with Naïve Bayes and Regression Tree learners.

### Relationships between Simpson index and best predictors

**Figure 8:**
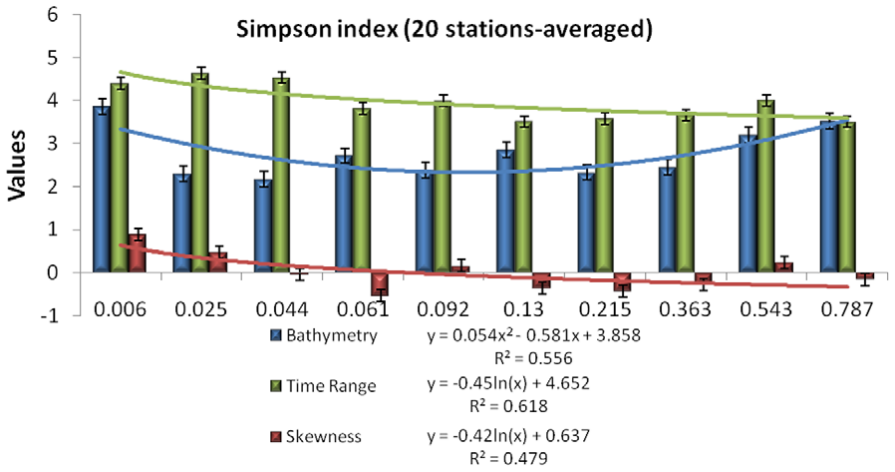
20 stations-averaged Simpson index in respect to the three 20 stations-averaged predictors highlighted by the Random Forest learner: bathymetry (light blue), time range (light green) and skewness (light red). Trendlines correspond to 3-degree polynomial fitting models and R2 stand for the coefficient of determination.

Even if values are low, Simpson index displayed significant correlations with bathymetry (Pearson correlation r = 0.1194, p = 0.0394*), time range (Spearman correlation ρ = −0.1771, p = 0.0021*), and skewness (Pearson correlation r = −0.0794, p = 0.1718; and Hoeffding correlation DH = 0.0210, p≤0.0001*). Simpson index therefore decreased with the vicinity of coastline, benthic waveform spreading as well as benthic waveform skewed to the right (visual support from Figure 2). The Simpson index and the three predictors were averaged per 20 stations in order to determine trends of the Simpson diversity across the three gradients (Figure 8). Highest values of bathymetry, time range and skewness (3.86 m, 4.39 ns and 0.92 ns, respectively) were associated with the lowest value of Simpson (0.006). Both time range and skewness gradients exhibited a constant decrease against Simpson index classes, as witnessed by their trendlines in the form of a natural logarithmic regression (R2 = 0.62 and R2 = 0.48, respectively). However, the bathymetry did not follow a clear decline against Simpson index, but fitting a 2-degree polynomial model properly matched their relationships (R2 = 0.56). The “synclinal” model relied upon deep bottoms linked with both the lowest and highest Simpson classes, as well as shallow bottoms related to medium Simpson classes.

### Predictive map

**Figure 9:**
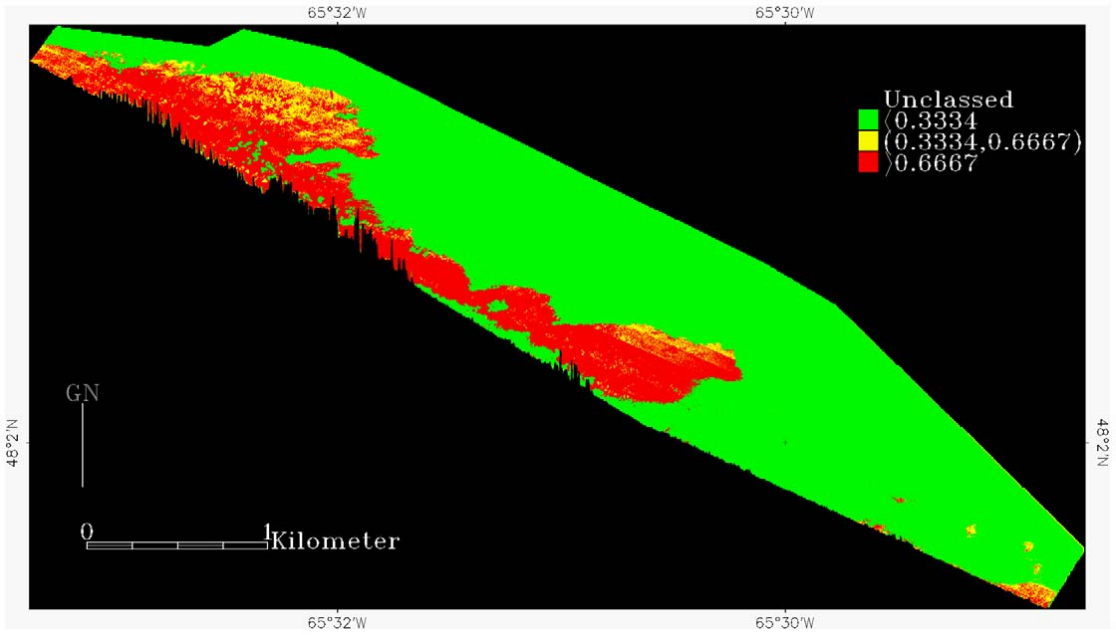
Map predicted epi-macrobenthic Simspon index (D) model for a neritic benthoscape of north of the Baie des Chaleurs (Québec, Canada) derived from the selected Random Forest Tree.

The modelled spatial distribution of epi-macrobenthic Simpson index highlighted both a diversified elongated offshore band to the west of the survey, scattered patches to the south, and a taxa-homogeneous area landward (Figure 9). The Simpson pattern of distribution was strongly influenced by bathymetry, as corroborated by its hierarchical status within the Random Forest splitting. Shallower-mapped pixels mostly appeared as belonging to the low class, except scarce and disseminated soundings. Otherwise, deeper-mapped pixels benefited much more from sample-balanced splitting, regarding the time range. While the high-labeled space units (i.e., low time range) were situated seaward, the medium-classified pixels (i.e., high time range) chiefly resulted over contours squeezed between low and high classes.

## Discussion

Explicitly comparing competitive machine learners enabled to select the Random Forest algorithm (Classification Accuracy: 0.69) and to capture an efficient combination of predictors of epi-macrobenthic Simpson diversity, despite their low Pearson's correlations. Through only three variables derived from bathymetric LiDAR waveform (bathymetry, time range and skewness), efficient predictive mapping of the biotic index was fully driven across turbid nearshore ecosystems.

### Revealing the depth and the structure of epi-macrobenthos habitat

The positive correlation linking Simpson index and bathymetry could be interpreted as a consequence of the hydrodynamic influence. Parallel to the coastline, a 50–100 m slice of sandy-gravely area was well nourished by meso- and microscale sedimentary dynamics (regional alongshore current and closeness to both an estuary and a tidal marsh), hindering an increase in the bottom structural complexity, correlated to the available amount of microhabitats. A little more seaward, substrata seemingly remained equivalent as in shallower areas regarding their niche-diversified properties, but actually lay on bedrock [23], which provided an augmented array of epi-macrobenthic refuges. Such added information stemmed from an acoustic survey, whose backscatter released meaningful products regarding the hardness/smoothness of the seabed. The evaluation of such parameters with LiDAR is an ongoing research, but has already been demonstrated at fine-scale (surface density of sand ripple correlated with a 532 nm LASER, [46]). Many biological and ecological processes within temperate coastal oceanic provinces are recognized to be driven by light, temperature, nutrients and currents, all of which are influenced by bathymetry [6], [12]. Although water depth did not directly act on benthic communities' structure and dynamics, bathymetry exerted an important control, or at least was a reliable proxy for proximal variables of Simpson diversity in shallow marine systems, considering the level of surrogacy inherent to these processes. Based upon the recurrent explanatory power of bathymetry among the wide range of environmental predictors available, the sea level rising could be assumed to induce landward shifts of controlling gradients, thus dramatic changes with which coastal and marine epi-macrobenthic organisms will have to cope.

**Figure 10:**
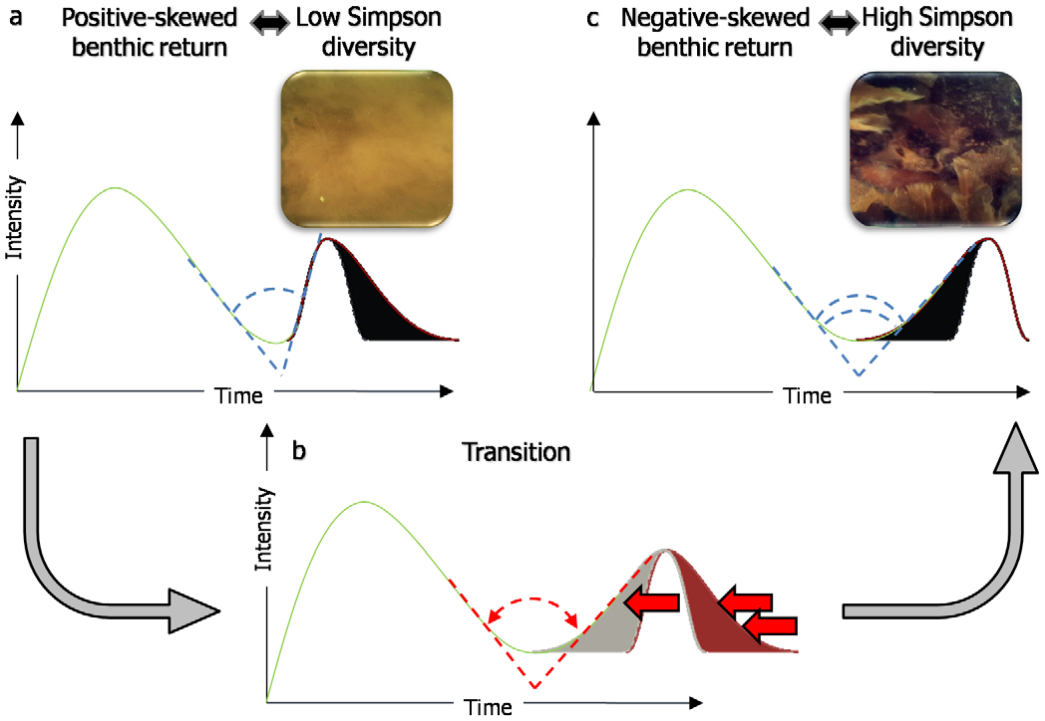
Hypothetical scenario explaining the evolution of the shape of the benthic waveform against the increase of Simpson diversity over seabed.

Otherwise, the negative relationships between Simpson index and skewness showed that the shape of the LiDAR benthic waveform could measure the biodiversity of such a shallow environment. The significant natural logarithmic fitting indicated that a low Simpson index matched a right-skewed benthic waveform distribution, while a high Simpson index corresponded to a left-skewed benthic waveform distribution. The pseudo-angle that could be drawn between the end part of the water column and the beginning part of the benthic return (cf. Figure 2) tended to be acute when photons related to the beginning of the benthic return were reflected evenly and within a relative short temporal window. This helped reveal the flatness of the benthic cover, hence the low Simpson diversity (Figure 10a). Conversely, the less acute the pseudo-angle was, the more widespread the distribution of the beginning of the benthic return was. This result was highlighted as a more complex benthic cover, providing a larger range of ecological niches, hence the high Simpson diversity (Figure 10b). Collin et al. [26] sought for assessing a bespoke “Habitat Rugosity” by means of LiDAR abilities and found a negative correlation (Pearson  = −0.17) between the designed index and the skewness stemmed from the LiDAR benthic return. This corroborates with our last result and supports the assumption that left/negative-skewed benthic return witnessed the vertical structural complexity of the benthic cover.

The negative relationship between Simpson index and time range implied that a Simpson-diversified benthic cover was related to a shortening of the benthic return distribution. Taking into account the link between Simpson index and skewness, the latter result could seem paradoxical as it is expected to see a temporal increase of the benthic return. However, the following scenario could suit the change of the benthic return against Simpson index:

a. low Simpson diversity over the seabed  = > photons were reflected in a relative short period due to the flatness of the seabed (i.e., acute angle between water column and benthic return), elongated tail of the waveform due to significant noise (positive-skewed benthic return)

b. increase of the Simpson diversity over the seabed  = > photons were reflected from and/or absorbed by an increasing suite of horizontal targets-strata inherent to surfaces of the structurally-complex seabed (i.e., less and less acute angle between water column and benthic return), shortening of the tail of the waveform due to the increasing reflection and absorption of photons before the peak of the benthic return (null- and negative-skewed benthic return)

c. high Simpson diversity over the seabed  = > photons were reflected from and/or absorbed by a wide range of horizontal targets-strata inherent to surfaces of the structurally-complex seabed (i.e., right or obtuse angle between water column and benthic return), absence of the tail of the waveform due to complete reflection and absorption of photons before the peak of the benthic return (negative-skewed benthic return).

The joint increase of the left-skewed benthic return and the Simpson index could be attributed to the increase in surface area of the substratum and the habitat-macrophytobenthos, which provide both an enhanced availability of ecological niches as well as specific microhabitats to demanding marine organisms [47]. By means of its capabilities to accurately detect seagrass/kelp canopy and stand structure as well as its ability to estimate above-bottom biomass, bathymetric LiDAR is emerging as a powerful tool to rapidly survey benthic diversity over nearshores ranging from the shoreline to threefold-Secchi-depth m. In agreement with well established ecological processes, areas with high epi-macrobenthic diversity were predicted for the most structurally-complex bottoms with high variability of both substrata and benthos, deduced by important left-skewed benthic return. Substratum type and dissimilarity or the bathymetric variability in the seascape were of secondary importance in this study.

### Modelling techniques

The Random Forest (RF) algorithm was able to satisfactorily fit relationships between epi-macrobenthic Simpson diversity and the environment. The separation process of the three-dimensional space, constituted by the three predictors, was sufficiently relevant to maximize the volume between the three Simpson classes, while minimizing the volume within each class, so that the other learners were outperformed. The RF highlighted an ecologically-meaningful threshold related to bathymetry. The 6-m isobath indeed embodied a boundary over which an embayment occurred and above which WE currents dominated. The relative protection of the embayment might have facilitated the settlement of an eelgrass (*Z. marina*) meadow and scattered macroalgae (*Chorda tomentosa*), thus the low Simpson index, whilst deeper contours, benefiting from nutrient supply driven by primary currents, might have provided niches for *Laminaria* spp., thus the high Simpson index spatially-modelled. This analysis testified the adequacy of using such a modelling technique to characterize ecological patterns and model them across significant areas.

As a summary of many decision trees, the RF found itself to be the best learner within the Classification And Regression Tree family, which is recognized to be highly efficient for predictive habitat modelling (see [4]). However, a novel CART approach, the Boosted Regression Tree (BRT), has repeatedly demonstrated its power of ecological modelling through terrestrial and marine studies [10], [19]. This machine learning technique grows a suite of regression trees (built from randomly-subset data) which predict the residuals inherent to the previous tree to consistently boost the overall predictive performance [48]. In the absence of this technique in Orange software, we could not have been able to integrate the BRT within comparisons of learners (lack of the evaluators and discretization). However, the Classification Accuracy (2/3 training and 1/3 validation) has been computed, for the sake of heuristics and completeness, with respect to the eight epi-macrobenthic indices using the Gaussian BRT implemented in R [49]: d = 0.45, A = 0.61, Log(A) = 0.63, D = 0.65, Log(D) = 0.65, H' = 0.54, Log(H'+1) = 0.56, mJ' = 0.67. The analysis of the best predictive model, i.e., the modified Pielou index, showed that the three best predictors were bathymetry, skewness and kurtosis, whose relative influence equaled 13.27%, 8.35% and 4.85%, respectively. RF and BRT consist of new statistical strategies for predictive habitat modelling which hold great promise to develop marine ecological theory.

### Implications to ecological theory

Albeit often neglected, there is an intimate relationship between ecological theory and method, either acquisition- or statistics-related [2], [4]. Biotic responses, investigated in this study, all represented diversity indices aiming at synthesizing the structural composition of benthic communities (e.g., the presence and abundance of benthic species). Modelling the spatial distribution of epi-macrobenthic heterogeneity boiled down to figure out the relationships between patterns of combined species responses against the environment. Niche theory assumes that collective properties, synthesized by Simpson diversity, do not show response patterns along environmental gradients [4], mostly because of differences in species growth forms inducing different response patterns [50]. However, such responses have been demonstrated at broad scale (>1 km) with vegetation communities in respect to indirect gradients [51], [52]. The novelty of our results evidenced for emphasizing curvilinear responses of marine communities at fine-scale (1 m) against an indirect gradient. The latter gradient, namely, bathymetry, contained direct (e.g., light) and resource (e.g., nutrient supply correlated with currents) gradients that led to robust predictive models. The curve depicting the relationship between Simpson index and bathymetry indicated that both low and high values of species heterogeneity matched with deeper bottoms in the study area, thus the “syncline-like” curve (Figure 8). Stronger currents below 6-m depth might have resulted in greater species heterogeneity than that of the embayment. Accounting for LiDAR bottom detection and light absorption by a dense canopy of *Laminaria* spp., the distribution of species heterogeneity could be assumed to be truncated at the observed upper (i.e., deeper) limits of the environmental predictor (i.e., bathymetry), since presence of *Laminaria* spp. has been testified over deeper bottoms in this region [23]. Knowing that species position along an environmental gradient has been shown to shape the response [53], the species heterogeneity distribution lying near its upper limits had to be somewhat biased. However, the bathymetric gradient was significantly steep given the difference between low depths of the embayment and deeper bottoms inherent to the seaward current-eroded slope. Being a distal variable encompassing the biodiversity response near its limits and characterized by a significant steep gradient (as advocated by [4]), the bathymetry can be considered as a successful predictor to those benthic communities.

The spatial scale at which photographs were acquired played a crucial role in those results. For instance, the species heterogeneity at high depths would have been profoundly reworked (trend to smooth differences) if the acquisition scale has been 10 m. The method of acquisition, besides constraining the scale, filtered out all cryptic and epiphytic macroorganisms. Large and wide thalli of macroalgae have precluded the observation of those organisms (e.g., asteroidea on thalli, echinoidea over seafloor, crustacea in crevices), when the video device projected the volume of algae onto the bottom surface. Both the size and behaviour of biological material strongly conditioned the computed Simpson index.

The exclusive use of LiDAR-derived environmental predictors constituted the main challenge of this modelling study, given the rapid data acquisition. Although the survey of biotic processes suffers from time-efficiency (hence cost-efficiency), it significantly contributes to the spatial patterning of benthic communities, as well as abiotic processes [3]. The optimization of data collecting concerning predation, competition, dispersal, recruitment and anthropogenic pressure needs greater consideration in order to incorporate them into statistical models of communities' distributions and to identify the “directness” of all these factors. The volitional omission of biotic processes into modelling did not entail low performance of the spatial prediction. We can assume that the RF learning process incorporated non-linear interactions between monitored environmental predictors and uncollected biotic processes. RF spatially modelled Simpson index into three discrete classes, impeding any continuous patterns between them. The classification of benthic communities into categorical types strongly fits with the traditional vision adopted by landscape ecologists [54]. However, most of the ecological variables can be deemed as fundamentally continuous, at least at the landscape scale. This prompts in handling concepts of communities as biotic-intricate superimpositions of continuous species responses to habitat suitability, instead of considering them as a mosaic of benthic “enclosed fields”. A more transparent practice may be to experimentally outline biotic and abiotic direct predictors, optimize the cost-efficiency of their collection (already optimized for LiDAR-derived geomorphic predictors), and analyze data with machine learners and Generalized Additive Models for Location, Scale and Shape (GAMLSS) [55], so that results can be readily interpreted from an ecological viewpoint.

### Conclusions

The combination of moderate ground-truth effort (underwater photographs), bathymetric LiDAR and the Random Forest learner allowed to robustly predict the spatial distribution of the Simpson diversity index related to epi-macrobenthic communities over a relatively turbid nearshore area. Bathymetry, skewness and time range were rapidly acquired by airborne LiDAR and had the greatest explanatory power to determine the Simpson diversity response. This species heterogeneity index showed an overall increase in respect to water depth (with a maximum value related to areas inherent to augmented hydrodynamics) and decreased with skewness and time range, which testified the increase of the structural complexity of the benthos/bottom. The degree of species heterogeneity was thereafter linked with the degree of their physical complexity.

This study displayed three major novelties within the realm of marine habitat modelling: predicting marine communities' diversity (rather than individual species), employing information from the bathymetric LiDAR full-waveform (not only the bathymetry and its related by-products), as well as confronting the state-of-the-art machine learners.

This work underpins that fully-exploited bathymetric LiDAR (not only bathymetrically-derived by-products), coupled with a powerful machine learner, is able to rapidly predict habitat characteristics at a spatial resolution relevant to epi-macrobenthos diversity ranging from clear to turbid waters. This method might serve both to nurture marine ecological theory and to manage areas with high species heterogeneity where navigation is hazardous and water clarity opaque to passive optical sensors.
